# Supplementary material for: Complexome profiling of the Chlamydomonas psb28 mutant reveals TEF5 as an early PSII assembly factor
Source: Plant Cell. 2025 Mar 17;37(6):koaf055. doi: 10.1093/plcell/koaf055 (PMC12164590; doi:10.1093/plcell/koaf055)
Supplement: koaf055_Supplementary_Data [file koaf055_supplementary_data.zip › psb28_interactive.zip!/plots/intensity/qualityPlots/peptides/_Cre14.g608800.t1.2.html]

### Cre14.g608800.t1.2

Trivial name:   
SeqCoverage [median]: 12.90  
Mass: 39.77 kDa  
  
 

| Sequence | MissedCleavage | Mass [Da] | wtIntensity | muIntensity | Score |
| --- | --- | --- | --- | --- | --- |
| AVAPQAPYNVVITGSTK | 0 | 1714.93 | 1009.25 | 654.97 | 63.09 |
| GLAVDVSAPGQAR | 0 | 1239.66 | 1106.88 | 578.50 | 79.15 |
| QLADFAAQELGR | 0 | 1317.67 | 0.00 | 477.94 | 72.01 |
|  |  |  | 2116.1 | 1711.4 |  |
